# Frequency of Depression in Patients with Type 2 Diabetes Mellitus and its Relationship with Glycemic Control and Diabetic Microvascular Complications

**DOI:** 10.7759/cureus.5145

**Published:** 2019-07-16

**Authors:** Sabira Sharif, Muhammad T Raza, Samsam Mushtaq, Bahjat Afreen, Bushra Azam Hashmi, Muhammad Hassaan Ali

**Affiliations:** 1 Medicine, Allama Iqbal Medical College/Jinnah Hospital, Lahore, PAK; 2 Medicine, Ameer-Ud-Din Medical College & Postgraduate Medical Institute, Lahore General Hospital, Lahore, PAK; 3 Ophthalmology, Allama Iqbal Medical College/Jinnah Hospital, Lahore, PAK

**Keywords:** diabetes mellitus, depression, frequency, complications, glycemic control, diabetic retinopathy

## Abstract

Introduction

Various clinical studies have reported that clinical depression is a common co-morbidity in patients with type 2 diabetes mellitus. Depression can badly affect the lifestyle of diabetic patients and impair the proper management of diabetes mellitus. Therefore, there exists a need to identify risk factors of depression in diabetic patients especially in relation to various clinical parameters, glycemic control and diabetic microvascular complications.

Materials and methods

It was a cross-sectional study conducted in a tertiary care hospital in Pakistan from August 2018 to April 2019. We recruited type 2 diabetic patients and measured their various clinical and hematological parameters. We evaluated depression using Patient Health Questionnaire (PHQ-9) and evaluated its relationship with glycemic control, duration of diabetes, fasting lipid profile and presence of various diabetic microvascular complications.

Results

There were 100 subjects in the study having a mean age 58.3 ± 12.4 (range: 36 - 71) years with a male to female ratio of 1:1.2. The mean duration of type 2 diabetes mellitus was 11.2 ± 9.2 years. The mean PHQ-9 score of the study population was 10.2 ± 8.1. The frequency of depression was found to be 40.0%. Depression was most frequently found in women and in patients between 40 to 60 years of age (60.0%). Depression was more common in patients with dyslipidemia (p-value = 0.0015). Patients with diabetic retinopathy, diabetic nephropathy and diabetic neuropathy were 3.8 times, 4.2 times and 2.1 times more likely to have clinical depression than the patients without these complications. Patients with glycated hemoglobin (HbA1c) worse than 7.5% had a significantly higher rate of depression than those whose HbA1c ranged from 6.5 - 7.5 % (p-value = 0.0028). Duration of diabetes mellitus did not significantly affect the frequency of depression in diabetic patients.

Conclusion

Depression is common in a large number of diabetic patients. Female gender, dyslipidemia, diabetic microvascular complications and impaired glycemic control are significantly associated with depression in diabetic patients.

## Introduction

Type 2 diabetes mellitus is one of the major causes of major end organ damage in the body including coronary artery disease, peripheral vascular disease, stroke, retinopathy and nephropathy and neuropathy [1]. The prevalence of diabetes is rapidly increasing and it is estimated that there will tremendous increase in diabetic population over next years [2]. Keeping this in mind, it is imperative that physicians employ all available resources to create awareness amongst diabetic patients to prevent its macrovascular and microvascular complications.

Various studies have shown a strong association of diabetes mellitus with major clinical depression [3]. Studies have been conducted to explore the two-directional association between diabetes and depression [4]. The evidence suggests that it is the depression which is a stronger causative factor of diabetes than the reverse [5]. Results of some meta-analyses have shown that diabetes almost doubles the risk of development of depression with greater occurrence in females than males [4,6]. The prevalence of depression in diabetes has been reported to be between 25 - 35% internationally [6].

Patients with diabetes need to put in extra efforts every day to match with the metabolic state of the individuals without diabetes, which significantly impacts the quality of life of the individual. It is the psychosocial behavior and mental status of a person with diabetes that affects the self-care and ultimately, long term glycemic control, the risk of developing long term complications, and quality of life [7]. Cause of depression in diabetes is multifactorial, ranging from increased pill count, associated obesity, end organ damage, increased substance abuse and other co-morbidities [8].

The productivity and growth of a country depend on the health-related quality of life of its people. The psychosocial factors have potent effects on physical health outcomes. Hence, having an assessment tool for evaluating the quality of life is a need of modern clinical evaluation. The relation between depression and diabetes is a vicious cycle resulting in adverse long-term glycemic control further worsening the risk of developing long term complications, end organ damage, poor quality of life, increased hospitalization, and even mortality. In spite of considerable data from the western world, the topic has received scant attention in the medical literature from Pakistan. Therefore, we conducted this study with the objectives to determine the frequency of depression among patients with type 2 diabetes mellitus and its relationship with glycemic control and diabetic microvascular complications.

## Materials and methods

It was a cross-sectional study conducted at the Department of Endocrinology, Jinnah Allama Iqbal Institute of Diabetes and Endocrinology (JAIDE), Jinnah Hospital, Lahore, Pakistan from August 2018 to April 2019. The study was conducted following principles of ethical medical practice as laid down in Declaration of Helsinki 2011 after obtaining approval of its synopsis from Ethical Review Board of Allama Iqbal Medical College/ Jinnah Hospital, Lahore, Pakistan. Patients were recruited in the study using non-probability purposive sampling. A sample size of 100 patients was calculated using World Health Organization (WHO) sample size calculator to estimate a proportion with 95% confidence level with the acceptable difference of 0.08 and assumed the proportion of depression to be 0.41 as reported in an earlier study [9].

Patients with type 2 diabetes mellitus for more than one year and age more than 35 years were included in the study. Patients were labeled to have type 2 diabetes mellitus if they fulfilled one or more of the following American Diabetic Association (ADA) criteria:

• Fasting plasma glucose ≥126 mg/dL (7 mmol/L). Fasting was defined as no caloric intake for at least eight hours;

• Symptoms of hyperglycemia and random venous plasma glucose ≥ 200 mg/dL (11.1 mmol/L);

• Abnormal oral glucose tolerance test (OGTT) defined as a plasma glucose ≥200 mg/dL (11.1 mmol/L) measured two hours after oral glucose load of 1.75 g/kg (maximum dose of 75 g);

• Glycated hemoglobin (HbA1c) ≥ 6.5%.

Patients with type 1 diabetes mellitus, or with history of gestational diabetes, history of severe co-morbid physical illness or cognitive impairment, any current psychiatric disorder other than depression, or history of substance abuse were excluded from the study.

Depression was evaluated using Patient Health Questionnaire-9 (PHQ-9). It is a multipurpose instrument for screening, diagnosing and assessing the severity of depression. It can be administered repeatedly which can reflect improvement or worsening of depression in response to treatment. PHQ-9 was scored from 0 - 27 and the severity of depression was labeled in a modified criterion mentioned below:

0 - 5: no depression

6 - 9: mild depression

10 - 14: moderate depression

> 15: severe depression

After taking informed consent from all the study participants, demographic and clinical data were recorded in a pre-designed questionnaire. Clinical data included biochemical parameters of diabetic control including HbA1c, fasting and post-prandial glucose readings, body mass index (BMI), fasting lipid profile, the renal profile including serum urea and creatinine, and detailed evaluations from consultant ophthalmologist (MHA), nephrologist and neurologist to evaluate any complications of diabetes mellitus. Patients who fulfilled the inclusion criteria were then referred to a consultant psychiatrist for treatment of depression.

Data analysis was performed using Statistical Package for Social Sciences (SPSS, IBM Statistics Inc, Chicago, IL, USA version 23.0). Mean and standard deviation were calculated for the quantitative variables like age, duration of diabetes mellitus, body mass index, HbA1c, and blood glucose levels. Frequencies and percentages were calculated for qualitative variables. Data were stratified for age, gender, BMI and duration of diabetes. Post-stratification chi-square test was used to assess the relationship between ordinal variables and depression whereas, Student’s t-test was used to assess the relationship between numerical variables with depression. A p-value ≤ 0.05 was considered statistically significant.

## Results

There were 100 subjects in the study with a mean age 58.3 ± 12.4 (range: 36 - 71) years with a male to female ratio of 1:1.2. The mean duration of type 2 diabetes mellitus was 11.2 ± 9.2 years. The mean BMI of the study population was 28.1 ± 6.1 Kg/m^2^. The mean HbA1c, fasting blood sugar level, serum cholesterol and serum creatinine were 8.1 ± 1.88%, 162 ± 51.2 mg/dL, 169 ± 53.2 mg/dL and 1.20 ± 0.56 mg/dL respectively. Table 1 shows details of other clinical parameters of the study population. The frequency of various diabetic complications in the study population is given in table 2.

**Table 1:** Demographic and Metabolic Profile of the Study Population SD: Standard Deviation; BMI: Body Mass Index; HDL: High Density Lipoprotein; LDL: Low Density Lipoprotein; HbA1c: glycated hemoglobin

Parameter	Mean ± SD
Demographic Variables	
Age	58.3 ± 12.4
BMI (Kg/m^2^)	28.1 ± 6.1
Men, n(%)	45 (45.0)
Women, n(%)	55 (55.0)
Metabolic Profile	
HbA1c (%)	8.1 ± 1.88
Fasting Blood Sugar (mg/dL)	162 ± 51.2
Serum Triglycerides (mg/dL)	148 ± 70.1
Serum Cholesterol (mg/dL)	169 ± 53.2
HDL (mg/dL)	39.1 ± 13.4
LDL (mg/dL)	101 ± 42.1
Hemoglobin (mg/dL)	13.2 ± 3.1
Serum Urea (mg/dL)	37.2 ± 22.0
Serum Creatinine (mg/dL)	1.20 ± 0.56

**Table 2:** Diabetic Complications in the Study Population

Complication of Diabetes Mellitus	Number of Patients (%)
Diabetic Retinopathy	22 (22.0)
Diabetic Nephropathy	14 (14.0)
Diabetic Neuropathy	16 (16.0)
Coronary Artery Disease	10 (10.0)
Cerebrovascular Accident/ Transient Ischemic Attack	7 (7.0)
Peripheral Vascular Disease	4 (4.0)

The mean PHQ-9 score of the study population was 10.2 ± 8.1. The frequency of depression was found to be 40.0%. We also correlated the effect of age on the frequency of depression and observed that the depression was most frequently found in patients between 40 to 60 years of age (60.0%) and less commonly in patients with age less than 40 years or greater than 60 years. Women were affected more frequently with depression as compared with men (13/45, 28.9% versus 25/55, 45.5%; p-value = 0.0012). Additionally, analysis of the severity of depression revealed that mild to moderate depression was more frequently found in diabetic patients than severe depression (35/40, 87.5% versus 5/40, 12.5%; p-value = 0.0041).

We also analyzed the association of depression with various clinical parameters of diabetic patients. It was observed that depression was more common in patients with dyslipidemia (p-value = 0.0015). Similarly, the fasting blood glucose level of patients with depression was significantly higher than the patients without depression (172 ± 49.2 mg/dL versus 151 ± 43.5 mg/dL, p-value = 0.001). Patients with diabetic retinopathy, diabetic nephropathy and diabetic neuropathy were 3.8 times, 4.2 times and 2.1 times more likely to have clinical depression than the patients without these complications (Table 3).

**Table 3:** Risk of Developing Depression in Association with Various Diabetic Microvascular Complications

Complication	Risk of Depression	Odd’s Ratio	Confidence Interval	p-value
Diabetic Retinopathy	3.8	3.83	1.11 – 10.4	0.00025
Diabetic Nephropathy	4.2	4.20	0.83 – 20.1	0.073
Diabetic Neuropathy	2.1	2.14	0.49 – 8.12	0.431

Lastly, patients with HbA1c worse than 7.5% had a significantly higher rate of depression than those whose HbA1c ranged from 6.5 - 7.5 % (p-value = 0.0028). Duration of diabetes mellitus did not significantly affect the frequency of depression in diabetic patients.

## Discussion

This study was conducted in the Pakistani population to determine the prevalence of depression among patients with type 2 diabetes mellitus and see the effect of various clinical parameters especially glycemic control and microvascular complications on depression. We found depression in 40% of the study population. Depression was more frequently found in women and in middle-aged patients between 40-60 years of age. Dyslipidemia, impaired fasting blood glucose level, poorer glycemic control (HbA1c >7.5%), and various diabetic microvascular complications increased the risk of depression in patients with type 2 diabetes mellitus.

In a study by Thour et al. the prevalence of depression was reported to be 41% [9]. Severe depression (PHQ score ≥15) was found in 3 (4%) subjects, moderate depression (PHQ score ≥10) in 7 (10%) subjects and mild depression was reported in 20 (27%) of subjects. The authors also reported that depression was more prevalent in patients with diabetic microvascular complications, higher fasting plasma glucose, hypertension, but the differences were not statistically significant [9]. Our study showed frequency of depression to be 41% which is in accordance with the results of Thour et al. We also observed the higher frequency of depression in females than males and frequency was even higher in housewives than working women. Earlier studies have also shown a similar pattern of depression in their cohort of patients [10,11]. Rahman et al. also reported that severe depression was clinically more in housewives than working women [11].

We also observed the relationship between depression and dyslipidemia. The patients with dyslipidemia were more prone to have clinical depression than the ones with normal fasting lipid profile. A study from Bahrain reported no effect of dyslipidemia on depression in diabetic patients which is in disagreement with the results of the current study [12]. Similarly, we also observed that patients with higher fasting blood sugar levels were more frequently affected with depression than the ones whose fasting sugar levels were relatively better controlled. Siddiqui et al. reported a similar trend and reported a statistically significant association of depression with fasting blood glucose level (χ^2^=5.16, p=0.022) [10]. This could be explained by the fact that diabetic patients generally monitor their blood sugar levels at home with portable glucometers. Raised sugar levels could potentially act as demotivating factors in these patients leading them to poorly control their sugar levels. This also highlights the need for continuous support and motivation for diabetic patients so that they are always driven to better control their blood sugar levels.

We could not find any significant effect of duration of diabetes mellitus on the frequency of depression. Similar results have earlier been reported by Siddiqui et al. and Raval et al. who also reported no association between duration of diabetes and depression [10,13]. On the contrary, some studies have reported a significant association between duration of diabetes and depression [11,14]. Glycemic control, however, was significantly related to the depression in diabetic patients. Patients with higher levels of HbA1c were linearly affected by depression. Our results contrast with some of the earlier studies which reported no association between glycemic control and depression [10]. Poor HbA1c in patients with depression also emphasizes the need to educate the patients about long-term, persistent control of diabetes mellitus instead of just one or two readings at one point of time. This long-term better control is required to prevent depression and other diabetic complications. We propose that poor glycemic control puts the diabetic patient at risk of developing various diabetic complications which in itself is an independent risk factor for depression as described below.

Our study investigated the effect of diabetic microvascular and macrovascular complications on depression in diabetic patients. We observed that diabetic nephropathy increased the risk of developing depression by 4.2 times followed by diabetic retinopathy (3.8 times) and diabetic neuropathy (2.1 times). Various studies have earlier reported a similar trend and shown a strong association between diabetic complications and depression [15]. Though some earlier studies have reported no association between diabetic complications and depression [14]. The discrepancy between these two kinds of studies could be attributed to the inclusion of diabetic patients with a relatively lower prevalence of co-existing diabetic complications in the later type of studies.

The findings of our study show that assessment of depression is an integral part of the complete assessment of a diabetic patient. American Diabetic Association has also recommended that depression should be screened and assessed in all patients with type 2 diabetes mellitus [16]. We also emphasize the role of the diabetic educator who needs to guide and educate diabetic patients and clarify any myths and false beliefs regarding diabetic management, especially in resource deficient developing countries. Patients diagnosed with depression mandate the need for referral to psychiatrists for early counseling and management so as to avoid a vicious cycle of non-compliance to anti-diabetic medications resulting in poor glycemic control and subsequent more depression [17-19]. A recent study has shown that depression increases mortality by 2.5 times in patients with depression as compared to people who are not depressed [20]. The mortality risk still remained very high despite adjustment for other effect modifiers including diabetic complications (hazard ratio of 1.76). So, though not the subject of the current study, we can anticipate adverse effects of undiagnosed and undermanaged depression in the form of decreased functioning, higher chances of suicide, and higher morbidity and mortality from all causes [20].

Our study had some limitations as well which included, first of all, its small sample size. We suggest future studies based on larger sample size including patients from various educational and socioeconomic backgrounds. Our study population consisted mainly of patients from an urban background. So, we suggest future research on people from rural background to see the differences between depression in rural and urban diabetic patients. Secondly, our study lacked a control group for comparison. Future studies are recommended to enroll a control group in the study for a better comparison of depression in diabetic patients with healthy controls. Lastly, a patient health questionnaire was used to measure depression only at one point of time. We did not follow up with the patients due to which we could not monitor glycemic control over a longer period of time and simultaneously correlate its effect on depression. We propose future studies following a prospective longitudinal design to study the correlation of glycemic control with depression. Future researchers can also study the efficacy of various anti-diabetic medications on the glycemic control and simultaneous relationship of HbA1c with depression. Lastly, we did not evaluate the effect of various types of diabetic retinopathy on depression in our study. Further studies can be planned to see the relationship between proliferative and non-proliferative diabetic retinopathy and clinical depression in diabetic patients.

## Conclusions

This study suggests that the prevalence of depression is much higher in Pakistani patients with type 2 diabetes mellitus. We recommend internists and endocrinologists to include evaluation of depression as part of routine detailed assessment of diabetic patients. In this regard, PHQ-9 questionnaire can prove to be a very useful and handy tool that can give reasonable idea about status of depression in the diabetic patient. Patients with higher scores on PHQ-9 could later be referred for in-depth psychiatric evaluation and simultaneously referred for comprehensive education about diabetes mellitus. Early diagnosis of diabetic complications and better control of glycemic control may prove to be very useful in limiting extent of depression in diabetic patients.
